# CORDAX web server: an online platform for the prediction and 3D visualization of aggregation motifs in protein sequences

**DOI:** 10.1093/bioinformatics/btae279

**Published:** 2024-04-25

**Authors:** Nikolaos Louros, Frederic Rousseau, Joost Schymkowitz

**Affiliations:** Switch Laboratory, VIB Center for Brain and Disease Research, VIB, 3000 Leuven, Belgium; Department of Cellular and Molecular Medicine, Switch Laboratory, KU Leuven, 3000 Leuven, Belgium; Switch Laboratory, VIB Center for AI & Computational Biology, VIB, 3000 Leuven, Belgium; Switch Laboratory, VIB Center for Brain and Disease Research, VIB, 3000 Leuven, Belgium; Department of Cellular and Molecular Medicine, Switch Laboratory, KU Leuven, 3000 Leuven, Belgium; Switch Laboratory, VIB Center for AI & Computational Biology, VIB, 3000 Leuven, Belgium; Switch Laboratory, VIB Center for Brain and Disease Research, VIB, 3000 Leuven, Belgium; Department of Cellular and Molecular Medicine, Switch Laboratory, KU Leuven, 3000 Leuven, Belgium; Switch Laboratory, VIB Center for AI & Computational Biology, VIB, 3000 Leuven, Belgium

## Abstract

**Motivation:**

Proteins, the molecular workhorses of biological systems, execute a multitude of critical functions dictated by their precise three-dimensional structures. In a complex and dynamic cellular environment, proteins can undergo misfolding, leading to the formation of aggregates that take up various forms, including amorphous and ordered aggregation in the shape of amyloid fibrils. This phenomenon is closely linked to a spectrum of widespread debilitating pathologies, such as Alzheimer’s disease, Parkinson’s disease, type-II diabetes, and several other proteinopathies, but also hampers the engineering of soluble agents, as in the case of antibody development. As such, the accurate prediction of aggregation propensity within protein sequences has become pivotal due to profound implications in understanding disease mechanisms, as well as in improving biotechnological and therapeutic applications.

**Results:**

We previously developed Cordax, a structure-based predictor that utilizes logistic regression to detect aggregation motifs in protein sequences based on their structural complementarity to the amyloid cross-beta architecture. Here, we present a dedicated web server interface for Cordax. This online platform combines several features including detailed scoring of sequence aggregation propensity, as well as 3D visualization with several customization options for topology models of the structural cores formed by predicted aggregation motifs. In addition, information is provided on experimentally determined aggregation-prone regions that exhibit sequence similarity to predicted motifs, scores, and links to other predictor outputs, as well as simultaneous predictions of relevant sequence propensities, such as solubility, hydrophobicity, and secondary structure propensity.

**Availability and implementation:**

The Cordax webserver is freely accessible at https://cordax.switchlab.org/.

## 1 Introduction

Proteins are the fundamental building blocks of life, playing pivotal roles in an array of biological processes. They are versatile molecules, executing functions ranging from catalyzing chemical reactions to providing structural support. However, the proper functioning of these biomolecules is inherently linked to their three-dimensional structure and stability (Dill and MacCallum 2012). In recent years, there has been a growing realization that misfolding and aggregation of proteins, including the formation of amyloid structures, are critical determinants of both debilitating diseases and valuable biotechnological applications (Chiti and Dobson 2017, Louros *et al.* 2023). Protein aggregation refers to the non-native, multimeric assembly of protein molecules, which often culminates in the formation of amyloid fibrils. These fibrils are characterized by their cross-β-sheet structure and have been implicated in a wide range of diseases including neurodegenerative disorders, such as Alzheimer’s, Parkinson’s, and Huntington’s disease, as well as localized or systemic amyloidosis, such as type-II diabetes or light-chain (AL) amyloidosis, respectively (Chiti and Dobson 2017, Buxbaum *et al.* 2022). The accumulation of misfolded protein aggregates in various tissues is a hallmark of these disorders and is associated with cellular dysfunction and organ failure. Conversely, in the field of biotechnology, protein aggregation and amyloid formation have emerged as both challenges and opportunities. Aggregation can reduce the yield and efficacy of recombinant protein production, affecting biopharmaceutical manufacturing processes and biotherapeutic product quality (Hamrang *et al.* 2013). Conversely, amyloid-like protein structures have found utility in the development of functional materials (Chakraborty *et al.* 2019, Jin *et al.* 2022), including nanotechnology, drug delivery or enzymatic catalysis (Ghosh *et al.* 2023, Yuan *et al.* 2023), and tissue engineering (Das *et al.* 2018), as well as a strategy for the targeted inactivation of hard-to-drug cellular factors related to diseases (Michiels *et al.* 2020, Janssen *et al.* 2023).

Consequently, it is essential to attain a comprehensive grasp of the factors that govern protein aggregation. The propensity of proteins to form amyloid structures is intrinsically encoded within their amino acid sequences (Tartaglia *et al.* 2008, Navarro and Ventura 2022). These sequences contain local motifs, historically referred to as “aggregation-prone regions” (APRs), “amyloid motifs” or “amyloidogenic determinants,” which have been demonstrated to actively facilitate the assembly of amyloid fibrils (Fernandez-Escamilla *et al.* 2004, Ventura *et al.* 2004, Teng and Eisenberg 2009). APRs are ubiquitously distributed throughout the vast spectrum of proteins (Sawaya *et al.* 2007, Teng and Eisenberg 2009, Goldschmidt *et al.* 2010, Louros *et al.* 2020, Sawaya *et al.* 2021). They are evolutionarily tied to the functional fold of soluble protein domains (Prabakaran *et al.* 2017, Langenberg *et al.* 2020), and are associated with the function of intrinsically disordered proteins (Santos *et al.* 2021), while also often acting as integral parts of transmembrane domains or protein-protein interaction interfaces (Castillo and Ventura 2009). In addition, short amyloid motifs have been shown to drive the formation of functional amyloid scaffolds, as for instance in the case of bacterial curli (Louros *et al.* 2016, Perov *et al.* 2019) or RHIMs, which form the necrosome complex or are employed by viruses attempting to hijack the same pathway (Mompeán *et al.* 2018, Baker *et al.* 2020). Numerous studies have elucidated the capacity of APRs to autonomously self-assemble into aggregates with characteristic amyloid-like morphologies when studied in isolation as peptide fragments (Sawaya *et al.* 2007, Guenther *et al.* 2018, Louros *et al.* 2020, Rawat *et al.* 2020). Their pivotal role in orchestrating the assembly of proteins is underscored by studies in which the introduction of APRs into proteins that typically do not aggregate induces their self-assembly (Ventura *et al.* 2002, Ivanova *et al.* 2004). Furthermore, mutational experiments have reinforced this link, demonstrating that altering specific residues within APRs with the intent of deactivating them results in the prevention of parental protein aggregation (Ventura *et al.* 2004, Teng and Eisenberg 2009, Guthertz *et al.* 2022). Recent research endeavours have also unveiled that APRs are capable of forming early intermediate species that are shared among various amyloid conformations of the same protein (Lövestam *et al*. 2024), known as polymorphs, form homotypic interfaces that act as protofilament contacts and establish common interactions that bolster the stability of fibril polymorphs extracted from the cerebral tissues of patients afflicted with various amyloid-related diseases (Sawaya *et al.* 2021, Louros *et al.* 2022, van der Kant *et al.* 2022, Mullapudi *et al.* 2023, Louros *et al.* 2024).

We recently developed a logistic regression model to predict amyloid propensity in protein sequences with high sensitivity and specificity (Louros *et al.* 2020). As a structure-based approach, this tool named Cordax was shown to uncouple protein aggregation propensity from traditional sequence propensities, such as hydrophobicity and solubility, thus, increasing its ability to detect less common APRs in protein sequences (Hughes *et al.* 2018, Santos *et al.* 2021) and to outperform current state-of-art software dedicated to detecting protein aggregation (Louros *et al.* 2020). Here, we report the development of a dedicated freely accessible webserver for Cordax that supports both the prediction and 3D visualization of predicted APRs in protein sequences.

## 2 Availability and implementation

**Figure 1.**
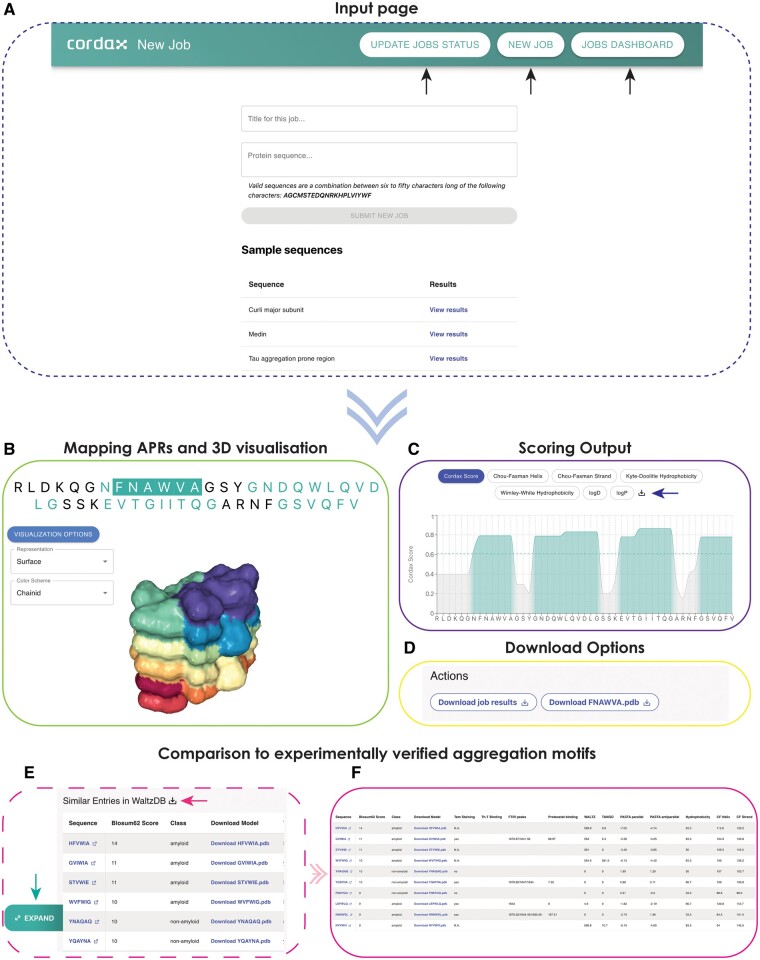
The Cordax web server interface. (A) Users can submit new jobs or track current and previous jobs through dedicated buttons on the webserver title page (indicated by arrows). Job submission requires a protein sequence as input, with an optional title. (B–F) Representative example of the information provided as output by the Cordax webserver. (B) The main interaction panel of the output page shows the query sequence, with predicted aggregation-prone regions highlighted. By selecting identified hexapeptide sequences, users can activate the 3D visualization plugin indicating the predicted steric zipper topology of the segment. (C) The scoring plot indicates by default the Cordax score per residue but can also be used to plot additional relevant sequence propensities. Users can access per-residue information through a box that appears by browsing over the query sequence shown on the x-axis. Access to the raw data is also provided through a download option (arrow). (D) Download options for information shown in (B and C) are also provided in the “Actions” submenu. (E) For predicted hexapeptides selected from the query sequence shown in (A), an interactive table is generated with experimentally determined aggregation-prone regions derived from WALTZ-DB (Louros *et al.* 2020) that are sorted based on sequence similarity scores. Information contained can be access using the download option at the top of the table (left-directed arrow). Using the expand option (down arrow), (F) a pop-up window appears for improved visualization of the table contents.

The Cordax web server is accessible to users online at https://cordax.switchlab.org/. This platform was designed and implemented using Netlify and is compatible with all devices and web browsers. While email registration is optional for users, it provides registered users the ability to maintain a personalized dashboard, enabling them to monitor the status of submitted tasks and access the outcomes of previous executions. The new job submission page, as well as the personalized dashboard, are both accessible through dedicated buttons that are permanently displayed on the web server title bar (Fig. 1A, arrows). Briefly, in the operational framework of Cordax, an input protein sequence is dissected into hexapeptides via a sliding window technique. Cordax employs the FoldX energy force field (Schymkowitz *et al.* 2005) to execute all-atom modelling of sequences against its structural database, as described previously (Louros *et al.* 2020), and the resulting free energies are converted into scores for each peptide fragment, using a recursive feature elimination algorithm and a logistic regression model trained against experimentally determined amyloid motifs (Louros *et al.* 2020). This process generates an amyloidogenic profile by assigning the highest score obtained for each residue within the input sequence (Cordax Score). A structural model that best represents the predicted amyloid fibril core topology is also selected for windows exceeding its scoring threshold (0.61). This operation is notably computationally intensive. However, computed energies are systematically recorded within an expanding database, facilitating subsequent retrieval. This engenders an efficient interface that circumvents redundant computational tasks for recurring sequence segments in future submissions. More information on the above, as well as a detailed description of the features offered through the webserver interface is provided in an “About” and “Help” page available online.

## 3 Features of the Cordax webserver interface

### 3.1 Main scoring display

The tool accepts simple protein sequences as input, with a minimum length of six residues and a maximum of 50 residues (Fig. 1A). All-atom modelling is a computationally intensive operation; hence, this length limitation has been set to expedite the webserver queue processing and to reduce output waiting times. The structural context of protein sequences is retained, as Cordax uses local sequence information to profile aggregation propensity. However, considering that it employs a hexapeptide sliding window, scoring of residues at the end of queries derived from longer sequences will derive only from the subset of hexapeptide windows included in the sequence query. To adjust for this, users can run sequence queries with overlapping ends, or alternatively use the standalone version of the tool that can be applied locally with no length constraints. Users are prompted to provide a title for each submitted job request, while completed processes can be accessed through the job dashboard.

Once accessed, each results page displays the query sequence on the top, with residues scoring higher than the Cordax threshold (0.61) (Louros *et al.* 2020) colored green (Fig. 1B). A graphical representation of the results shown at the bottom of the output page better illustrates this. Specifically, this interactive plot contains the amino acid query sequence on the *x*-axis, while alternative options are available to the user for display on the *y*-axis (Fig. 1C). Starting with the Cordax scoring as the default representation, by hovering over the query sequence a box appears labeling both individual residues, their corresponding Cordax aggregation scores, and the defined threshold of prediction. The latter is also shown with a dashed green light. The same interactive features are available for additional sequence properties that can be selected by the user and displayed on the interactive plot (Fig. 1C). For secondary structure propensity, we used the Chou–Fasman empirical technique (Chou and Fasman 1974). Sequence hydrophobicity is calculated based on two different scales, namely the Kyte–Doolittle (Kyte and Doolittle 1982) and the Wimley–White scale which holds considerable importance as it considers the combined contributions of both the peptide bonds and the sidechains in absolute values, providing a direct and empirical foundation based on experimentally determined values for the transfer free energies of polypeptides (Wimley and White 1996). Finally, considering the ability of Cordax to predict with high accuracy aggregation-prone sequence segments of higher solubility, we have included per residue calculations of partition coefficients calculated using PlogP, a method that calculates peptide coefficients by a residue-addition method and also considers blocked termini, as well as partition as a function of the pH (ionizable and non-ionizable) (Tao *et al.* 1999). A download option is also available for obtaining and analyzing the data presented in the interactive plot locally.

### 3.2 Modelling the structural topology of predicted aggregation-prone regions

The sequence presented at the top of the output page is interactive, whereby individual predicted residues can be engaged by a user. This interaction serves to illuminate the protein sequence segments that score above the threshold. Clicking on predicted residues highlights the hexapeptide window of prediction starting with this residue in position 1. If this window scores above the threshold of prediction, this selection concurrently activates a graphical plugin interface situated beneath the query sequence (Fig. 1B). Within this graphical interface, various modes for representing the structural topology of selected hexapeptides that surpass the Cordax aggregation propensity threshold are supported. These modes encompass options such as cartoon, ball and stick, ribbon, space-fill models, and surface representations, among others. Furthermore, a range of distinctive color themes are provided predicated on diverse properties, including chain ID, atom and residue types, and hydrophobicity (Fig. 1B).

### 3.3 Comparison to peptides with experimentally determined amyloid-forming properties

For each hexapeptide region selected from the displayed query sequence, an adjacent right panel becomes active, offering several supplementary features. Primarily, users are provided with the option to download specific content at the top of this panel (Fig. 1D). This includes the Cordax scoring files in the .csv file format and the predicted structural topology in Protein Data Bank (wwPDB Consortium 2019) file format (.pdb files) for windows scoring above the threshold. Simultaneously, upon the selection of a hexapeptide, an interactive table is displayed on the right panel (Fig. 1E and F). This table, which can be expanded for improved visualization by moving the cursor over the table and selecting an expansion button option appearing on the left, enumerates peptide sequences that correspond to entries within WALTZ-DB 2.0, currently the largest openly accessible repository of peptides with experimentally ascertained amyloidogenic properties (Louros *et al.* 2020). The sequences are organized based on their sequence similarity to the selected hexapeptide, calculated using the Blosum62 matrix. This table further provides valuable data concerning the employed experimental techniques used to determine the aggregation properties of each peptide entry. This includes experimental validation obtained from diverse methodologies like Transmission electron microscopy (TEM), Fourier-Transform infrared spectroscopy (FTIR), and the binding of various fluorescence aggregation reporter dyes (such as Thioflavin-T and Proteostat binding). In addition, aggregation propensity prediction scores are listed, generated by other specialized high-specificity tools, such as WALTZ (Maurer-Stroh *et al.* 2010), TANGO (Fernandez-Escamilla *et al.* 2004), and PASTA 2.0 (Walsh *et al.* 2014) (for both parallel and anti-parallel orientation predictions, as described). Notably, the data presented in the interactive table can be downloaded locally using an option at the top of the table, and predicted topologies of the sequences can be downloaded in a .pdb format through a dedicated column containing links. Finally, each sequence presented in the table is hyperlinked, enabling direct access to the corresponding peptide entry within WALTZ-DB (Fig. 1E and F). This facilitates users in acquiring supplementary and pertinent information. Such information encompasses details regarding the source proteins from which the peptide matches originate and are initially analyzed within WALTZ-DB, denoted by their Uniprot identifiers (The UniProt Consortium 2023), along with their respective positions in the identified protein sequence. Additionally, users can access a comprehensive breakdown of individual energy components and the topological models predicted by Cordax for the specific peptide sequence entry. Moreover, these links provide access to additional aggregation prediction algorithms, such as Zipper-DB 3D-profiling method (Thompson *et al.* 2006) and Aggrescan (Conchillo-Solé *et al.* 2007). Lastly, they can access a visual representation of the experimental evidence confirming the aggregation propensity listed in the initial table.
